# Conscious sedation: is this provision equitable? Analysis of sedation services provided within primary dental care in England, 2012–2014

**DOI:** 10.1038/bdjopen.2016.2

**Published:** 2016-02-26

**Authors:** Kristina L Wanyonyi, Sandra White, Jennifer E Gallagher

**Affiliations:** 1 Population and Patient Health, King’s College London Dental Institute, London, UK

## Abstract

**Aim:**

Patients receiving primary dental care may occasionally require conscious sedation as an adjunct to care. It is one of a range of options to support anxious patients or those undergoing difficult procedures. The aim of this study was to examine patterns of conscious sedation within primary dental care in relation to patient demography, deprivation status, geography (local authority, region) and type of care (Band) within England to examine equity in distribution of service provision.

**Materials and Methods:**

Descriptive analysis of cross-sectional primary dental care data, obtained from national claims held by the National Health Service (NHS) Business Services Authority, on patients who had received one or more courses of care involving sedation.

**Results:**

Just under 137,000 episodes of care involving sedation are provided for over 120,000 patients per year, the majority of which are for adults. Four out of ten (41%) patients were children, with 6–12-year-olds forming the largest group; 6% were aged under six years. Eleven per cent of patients had more than one course of care involving sedation, with adults aged 25–34 years having the highest rate: 1.17 (s.d.: 0.887) in 2012/2013 and 1.16 (s.d.: 0.724) in 2013/2014. There was a clear social gradient, whereby the most deprived quintile had the highest volume of patients that had received sedation at least once in primary dental care in both years (31.5%). Whilst there was a clear social gradient amongst children and young adults who received sedation, the gradient flattened among middle-aged and was flat amongst older adults. The majority of courses of care involving sedation were associated with Band 2 claims for care (88.6% in 2012/2013; 88.8 in 2013/2014). Whilst one or more patients in all higher tier local authorities received care involving sedation, there were marked geographic inequalities.

**Discussion:**

Patients receive sedation in support of NHS primary dental care across the life course and social spectrum. Whilst the pattern of uptake of care parallels the social gradient in younger age groups overall, there are clear geographical inequalities in provision. As sedation is only one of a series of adjuncts to care which may be provided across different sectors of the health system, a wider systems analysis should be undertaken as the findings raise important issues about equitable access to appropriate care. Furthermore, there should be a greater emphasis on prevention to reduce the need for care. The implications for child oral health, access and quality are discussed.

## Introduction

Sedation is an important adjunct to support the provision of dentistry for patients whose co-operation, anxiety or health, requires this service, including those require assistance because of a difficult or lengthy dental procedure. It is part of a continuum of management approaches, from behavioural therapy through to general anaesthetic available to support dental team members in the provision of contemporary dental care, where local anaesthetic alone is insufficient.^1^ Nationally, the majority of dentistry is provided in primary-care settings, where sedation is delivered by dentists directly or with the support of a sedationist.

Oral health nationally has improved markedly over the past four to five decades;^2,3^ however, dental diseases remain prevalent, and a majority of the population requires dental treatment at some stage in their lives.^4^ For a small cohort, their needs are extensive, and there is clear evidence that needs for care are socially defined. Adults, and children, from lower social groups are more likely to have dental caries, attend irregularly for care, and receive remedial action in the form of tooth extraction.^5,6^ For young children, who may be pre-cooperative, this often involves admission to hospital for extractions under general anaesthesia (GA) or sedation.^7^ For others, anxiety about dental care means that sedation is required alongside local anaesthesia to enable dental care to be delivered; and a third group may require sedation just to assist with a complex procedure.^8^

The data from primary dental care in England indicate that there is currently a static overall volume of sedation provided each year in primary dental care under the present National Health Service (NHS) system, whereby sedation is commissioned as ‘Part 9, Additional services, Advanced mandatory services’ in Personal Dental Services Agreements and General Dental Services Contracts, within the NHS.^9^ Under these arrangements there are just over 136,000 courses of treatment in primary dental care involving the use of conscious sedation across England.^10^ The data on sedation provided to support care in hospitals are combined with those for GA and, thus, the level of sedation services is not available separately. Sedation services are also available in conjunction with private dental care and these data are not available for England; however, a recent report in Northern Ireland suggests that just over half of sedation services in the province are provided in the private sector.^11^

Within the last decade, an Index of Sedation Need has been developed, and tested, within England.^12–16^ Research using this index suggests that the need for sedation services may be higher than the level of current provision, and that whilst most patients receiving sedation do require this support, a minority may not;^12,14,15^ however, there has been no national consideration of the need for sedation need and services.

An intercollegiate working group, the Intercollegiate Advisory Committee for Sedation, has recently highlighted the importance of care pathways and processes to ensure quality care is delivered for all patients.^1^ This report outlines the importance of access to a range of supportive approaches from ‘behaviour management’ through access to ‘behavioural therapies’ such as cognitive behavioural therapy, as well as conscious sedation and general anaesthesia services.^1^ The provision of dental care within the National Health Service in England is moving towards the establishment of appropriate-care pathways;^17,18^ defined as ‘a methodology for the mutual decision-making and organisation of care for a well-defined group of patients during a well-defined period’.^19^ Furthermore, important evidence of the role of cognitive behavioural therapy in dental care is emerging as a route to manage anxiety effectively.^20,21^

In this context where there is a limited volume of care commissioned, and alternatives are emerging, it is important to understand who is receiving sedation as an adjunct to care, the age and social profile of patients, the geographic distribution across NHS regions and authorities and the nature of service provision in relation to the NHS Bands of care, if population needs are to be met equitably.

The aim of this research was to examine patterns of conscious sedation within primary dental care in relation to patient demography, deprivation status, geography (local authority and region) and type of care (Band) within England to ascertain the equity in distribution of service provision.

## Materials and Methods

Data on patients that had received conscious sedation under state-funded (NHS) primary dental care in England for the years 2012/2013 and 2013/2014 were obtained following a request to the NHS Business Service Authority. These data were cross-sectional and included variables related to patients’ age band, region, higher tier local authority, postcode and the number and type (NHS Band: 1, 2, 3 and urgent) of courses of care under conscious sedation for dental treatment. Urgent treatment covers examination, X-rays and treatments such as dressings, re-cementing crowns and up to two tooth extractions and one tooth restorations. Band 1 involves assessment of a patient, which includes diagnosis, treatment planning and maintenance, examination, X-rays, scale and polish, preventative work and minor changes to dentures. Band 2 constitutes any Band 1 items plus treatment that does not involve laboratory work, for example, tooth restoration, root canal treatment, tooth extraction and periodontal treatment. Band 3 involves complex treatment that includes a laboratory element, for example, bridges, crowns and dentures, in addition to any Band 1 or 2 level of care.

The service data were augmented to include the quintile of deprivation for each patient based on area of residence and local authority. This two-stage process involved, first, patient postcodes being converted into the relevant lower layer super output area (LSOA). LSOAs are small areas of residence in England of relatively even size containing ~1,500 people, and used to develop the Index of Multiple Deprivation and supplementary indices to measure deprivation.^22^ This conversion was undertaken using GeoConvert tool on the Office of National Statistics website.^23^ There are a total of 32,482 LSOAs ranked by Index of Multiple Deprivation (IMD) 2010 in England. To obtain the local authority name, GeoConvert was used to convert LSOA to local authority code; these were further merged with local authority name spreadsheets from the Office of National Statistics. Second, quintile of deprivation was determined using the IMD 2010, which was obtained by merging spread sheet data by LSOA, with details of patients’ IMD 2010 from the Department for Communities and Local Government.

The data set variables having been determined, patient age and deprivation quintile were described by year, followed by an analysis of the proportion of sedation treatments under each NHS Band of care, as well as geographic distribution by region and local anaesthesia.

## Results

### Volume of care

Whilst there were 136,618 courses of NHS care involving sedation in England in 2012/2013 and 136,263 in 2013/2014, this represented care for 120,035 and 120,468 patients, respectively, based on FP17 claim forms returned to the NHS Business Service Authority. Each FP17 constitutes one completed/closed course of care under any one of the four NHS Bands of treatment.^24^ The number of courses of care involving conscious sedation was similar across both years but 0.3% lower in 2013/2014 (Table 1).

### Age profile of patients

Patients spanned the life course with the lowest proportions in the extremes of age (0–2 and ⩾75 year age bands) across both NHS years (Table 1). Children represented just over 40% of patients. The 6–12-year age band involved the highest proportion of patients that had received at least one course of care under sedation, with 23.7% of the patients in 2012/2013 and 24.3% in 2013/2014. Around 6% were aged under 6 years, and it is notable that although small in number, there were children under 2 years of age receiving a course of care involving sedation.

### Deprivation profile of patients

Over 97% of patient postcodes were converted into IMD scores with only 2% (*n*=2,391 in 2012/2013) and 2.4% (*n*=3,011 in 2013/2014), respectively, not obtained due to unrecognisable postcodes. Deprivation scores were missing for 3,134 (2.6%) patients in 2012/2013 and 3,182 (3.1%) in 2013/14;, and for these patients the rate of courses of care per patient involving sedation per patient was high: 1.44 in 2012/2013 and 1.33 in 2013/2014, indicating a higher level of repeat courses of care involving sedation.

**Figure 1:**
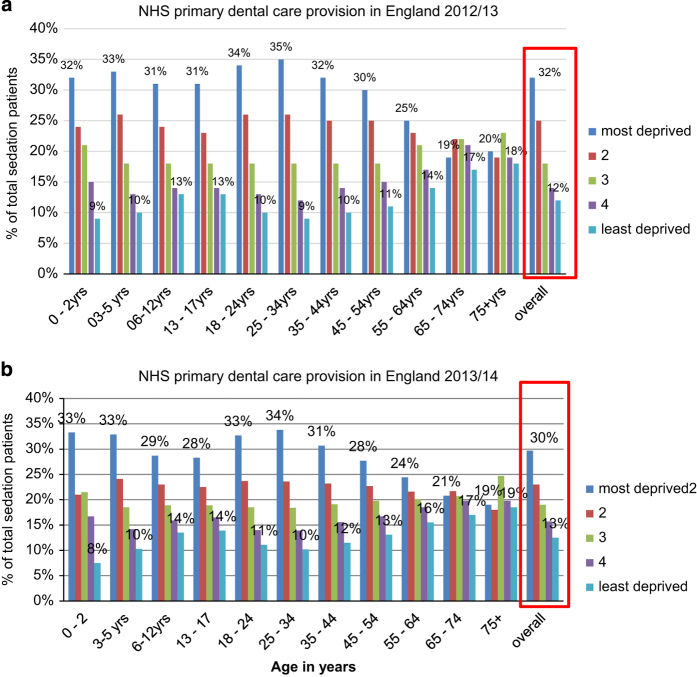
Patients receiving sedation by age band and quintile of deprivation, (**a**) 2012/2013 and (**b**) 2013/2014.

For the patients with identifiable deprivation scores, the age distribution of was similar to the overall sample (Figure 1). For those with missing IMD, the highest proportions were 25–34-year-olds in 2012/13 (24.4%), and 6–12-year-olds in 2013/2014 (24.5%). There was a clear social gradient between deprivation status and patients receiving sedation during a course of care; the most deprived quintile having the highest volume of patients receiving sedation, which represented 31.5% of the total patients in each year, whilst the least deprived quintile represented 11.5% of patients in 2012/2013 and 11.7% in 2013/2014. The social gradient was particularly marked amongst children and younger adults; however, amongst middle-aged and older adults (65–74-year-olds; ⩾75 years), there were similar levels across the quintiles of deprivation.

### Courses of care involving sedation

**Figure 2:**
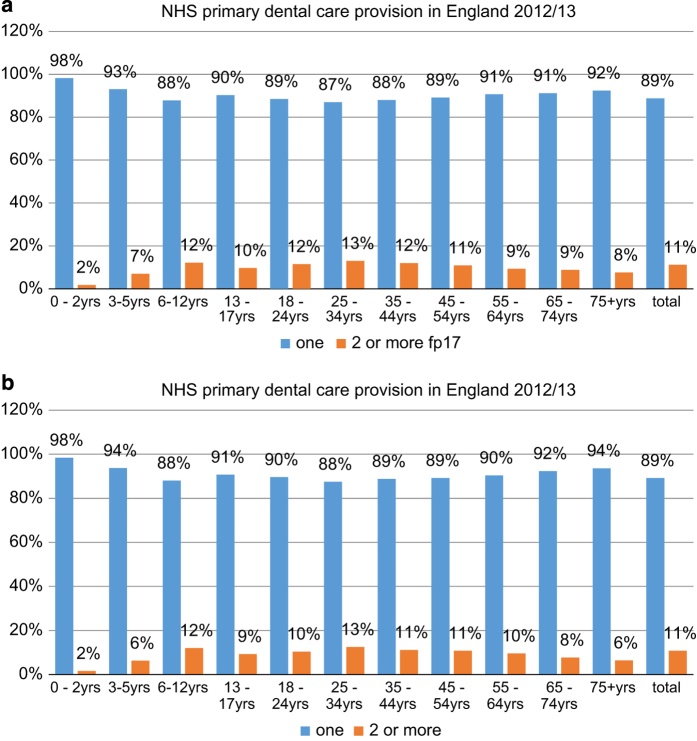
Patients with one or more courses of care involving sedation by age band, (**a**) 2012/2013 and (**b**) 2013/2014.

Overall, one in nine patients had a repeat course of care involving the use of sedation (11.2% in 2013/2014; 10.8% in 2013/2014) as shown in Figure 2; thus the rate was 1.14 (s.d.: 0.703) in and 1.13 (s.d.: 0.605), respectively (Table 2). Patients aged 25–34 years had the highest recorded rate of courses involving sedation across both years, with an average of 1.17 (s.d.: 0.889) courses in 2012/2013 and 1.16 (s.d.: 0.726) in 2013/2014. In relation to deprivation, the highest rate was amongst the most deprived quintile in both years: 1.16 (s.d.: 0.464) and 1.15 (s.d.: 0.305).

### Type of care (NHS treatment Band of care)

Analysis of courses of care involving sedation by NHS Band of treatment suggests the following patterns as presented in Table 3. First, the rate of Band 1 courses of care involving sedation was highest amongst 0–2-year-olds (0.12 in 2012/2013 and 0.26 in 2013/2014). Second, Band 2 courses of care were high across all age bands, but the rate of Band 2 courses involving sedation was highest amongst 6–12-year-olds (1.09 in 2012/2013 and 1.08 in 2013/2014).

**Figure 3:**
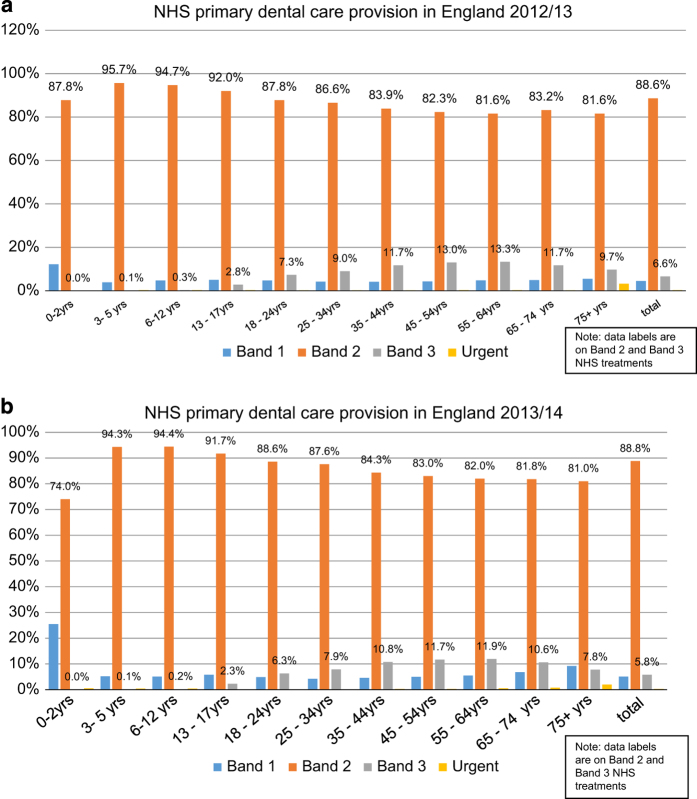
Courses of dental care involving sedation by treatment band and age, (**a**) 2012/2013 and (**b**) 2013/2014.

In Figure 3 the courses of care involving sedation by NHS Band of treatment, as a proportion of the total course of care, in each deprivation quintile are presented. The findings suggest that although Band 2 treatments are by far the highest in proportion for all age bands, in the middle-aged and older adults’ Band 3 courses of care are higher than other age-groups. In addition, older adults (⩾75 years) had a higher proportion of urgent care (8.9% for 2012/2013 and 7.5% for 2013/2014) than other age bands.

### Geographic distribution: local authority and region

**Figure 4:**
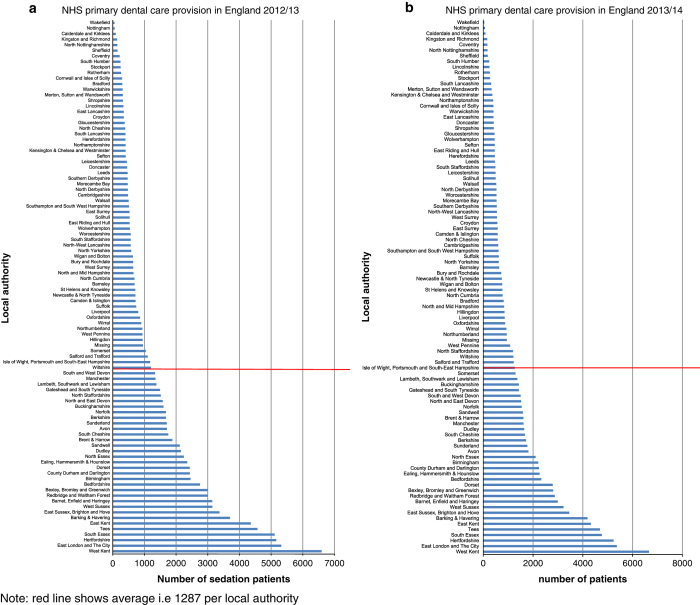
Sedation patients by Upper Tier Local authority of residence (**a**) 2012/2013 and (**b**) 2013/2014. Note: red line shows average, i.e., 1,287 per local authority.

All 95 upper tier local authorities had at least one resident person receive a sedation treatment in both years. Figure 4 shows the number of sedation patients by local authority, with the average rate per claims being 1,285 in the year 2012/2013 and 1,287 in 2013/2014. The findings suggest that 33–34 authorities had a higher than average number of patients who had received sedation (2012/2013 and 2013/2014). In both years, West Kent had the highest volume, accounting for 5% of NHS primary dental care claims.

**Figure 5:**
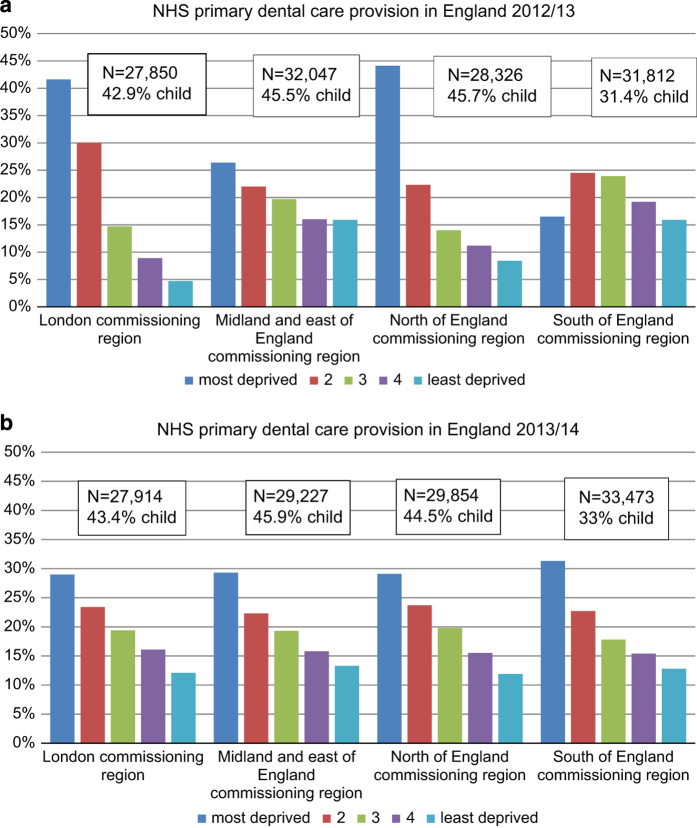
Region of patient residence and deprivation status, of patients receiving sedation within a course of care, (**a**) 2012/13 and (**b**) 2013/14.

The findings, by region, consistently highlight the social gradient in uptake of care involving sedation across all but South of England in 2012/2013 (Figure 5). The volume of care is broadly similar across three of the four regions with South of England having the highest level of provision; interestingly, this is also the region where children’s care is lowest. Whilst adults form 59% of overall patients, in the South of England they comprise 67–68%.

## Discussion

First, analysis of NHS activity data reveals a marked social gradient in the uptake of conscious sedation within primary dental care, as demonstrated by deprivation based on residential postcode, which is most pronounced amongst children and young people.^3^ Second, it highlights possible geographic inequalities in access at higher tier local authority and possibly regional level. Third, it raises important quality issues in relation to children’s dental care, both in relation to caries management and the provision of sedation.

### Social gradient

The social gradient in the uptake of conscious sedation within primary dental care, broadly parallels the social gradient in dental caries experience,^3,25^ and hospital admissions for extractions,^26,27^ in the population. These findings clearly reveal that patients with postcodes in the most deprived quintile were most likely to receive care involving sedation in primary dental care, which amounted to 31.5% of patients across both financial years; they also had the highest rate of courses of care per patient involving sedation across both years examined: (1.16 (s.d.: 0.464) and 1.15 (s.d.: 0.305), respectively).

Anxiety levels, which are a major influencing factor in relation to sedation need,^12,14^ are also reported as highest in lower social groups;^28^ 14% of participants from routine and manual occupations reported extreme dental anxiety, compared with 10% of managerial and professional and 12% of intermediate occupations, in the last national adult dental health survey.^5^ In addition, children from socially deprived groups have also been shown to have more extensive decay experience,^3,6^ which is likely to result in the need for more intensive work that could be undertaken involving sedation.

Disease management is affected by late dental attendance, also associated with higher deprivation, and results in higher rates of extractions as evidenced by the findings of national surveys,^3^ and local research.^3,25,29^ Mejia *et al.*,^30^ in an Australian wide survey showed that oral health inequalities were more apparent in measures that reflected disease management as opposed to outcome measures of disease experience. Furthermore, whilst a social gradient in dental caries experience (decayed, missing and filled teeth), it is particularly notable in the ‘missing’ and ‘untreated decay’ subcategories; thus, lower social position was associated with the nature of dental treatment reported to have been received in the previous year.^31,32^

Interestingly, the relationship changed with increasing age, as among the middle-aged and older age bands the gradient flattens, most notably, in 65–74-year-olds. This may be associated with reduced levels of NHS service use and an absence of any correlation between deprivation and NHS service uptake,^33^ higher levels of edentulousness,^34,35^ and higher levels of private dental care.^5^

Within deprivation quintiles the distribution of NHS Bands of care was similar, with Band 2 highest and urgent courses of care the lowest. Patients with missing IMD had a higher proportion of urgent courses of care compared with those with IMD score 0.06 compared with 0.03, which may be associated with more chaotic, or mobile, lifestyles.

What these data clearly suggest is that overall many of those who most need dental care do manage to access NHS care and support, when required; however, the geographic findings, examined below, suggest that this might not be the case in all parts of the country.

### Geographic inequalities

There is wide geographic variation in sedation provision within NHS primary dental care, which relates to supply and may also relate to historical inequity in access; a pattern that has continued with commissioned care. Whilst there may be equity of access, for some it must be remembered that activity reflects historic service provision and geographic inequity of access for many in England, similar to uptake of routine care, where the inverse care law is evident.^36^ It would, therefore, have been expected that higher levels of sedation would have been provided in the north of England where oral health needs are highest.^37^ Furthermore, as already suggested, this is not the whole picture of need as many patients, notably children, receive admissions to hospital for care under GA or sedation, whilst others (mainly adults) may access sedation services privately, as with Northern Ireland, hence, the level of care overall may not be insubstantial.^38^

Is the right level of service commissioned within the NHS primary dental care system and is it provided in the correct places? How does the sedation service relate to need and the level of hospital provision? To answer these questions a ‘whole systems approach’ should be taken to examine the level of need nationally to ensure that there is equitable access for those who need it across the new unified NHS system, and locally, across service providers in so far as this is possible. It is important to determine what services are available to support clinicians and patients when care cannot be provided under local anaesthesia across hospital or primary care, both NHS and private systems. And, most importantly, to reflect across types of care from cognitive behavioural therapy^21^ to GA, with a care pathway approach being used to ensure that dental professionals have access to the necessary support services for their patients across health-care organisations.^18^ This could involve testing the use of Index of Sedation Need as a tool to assess need.^12–15^ Without providers assessing, and reporting, the need for sedation, commissioners will be unable to audit the capacity of service activity to meet need. Together these actions will support policy makers and practitioners to ensure that patients receive appropriate care at each stage of their dental journey. In support of this, planners and commissioners of services should audit the provision of these adjuncts to care, by assessing access, quality and capacity to meet need. The systems approach would need to examine the need of the people within the system, and ensure sufficient capacity of services for those in greatest need. In order to facilitate access to appropriate care, information on local access to the full range of adjuncts should be clearly available for patients and dental care providers, with the needs of the patients being paramount in a patient-centred health-care system.^39^

### Disease management and conscious sedation in children

These findings act as a stark challenge to the nature of oral and dental care for children and sedation services for children. The largest age group that received sedation, at least once across both time periods, was 6–12-year-olds accounting for almost one quarter of cases; this is similar to the profile of NHS hospital admissions, where the majority of children gaining admission for removal of teeth are between the ages of 5 and 14 years.^40^

Children are more likely to be considered for sedation if they are young (pre-cooperative) or if they have extensive decay be considered appropriate for surgical or restorative management of disease at this age, the extent of which is too great to manage under local anaesthetic alone. Whilst the sedation data from primary dental care are not linked to the treatment details, the high level of Band 2 courses suggests that these children will most likely be receiving extractions and/or restorative treatment. Whilst it was surprising to find Band 1 courses of care, this may be merely an administrative error; it was highest amongst 0–2-year-olds, where 11.9% of all sedation courses of care were Band 1 compared with all other age bands (<6%). It raises the question: why are 0–2-year-olds having sedation? Presumably they are pre-cooperative? And if they are only having a Band 1 episode then it suggests that the sedation may be for assessment and diagnosis, rather than care or treatment is not possible. Are some of these children then referred to hospital for a GA? Dental practices should be encouraged to look at their data carefully and audit what is happening in younger children in their care. Furthermore, the nature of sedation provided now warrants close scrutiny, particularly for children under 12 years of age. The recent paper by Coulthard *et al.,* ^41^ clearly demonstrates that whilst the majority of children in this age bracket are reported to have inhalation sedation, in line with recent guidance,^1^ there is a significant proportion having single or multiple i.v. drug therapies. Now that Intercollegiate Advisory Committee for Sedation has published detailed guidance on conscious sedation in dentistry, it is important that providers and commissioners work to ensure that those who most need sedation services receive care in line with the intercollegiate guidance, particularly those under 12 years of age.^1^

Given the increasing emphasis on prevention,^42,43^ and the evidence base for other non-surgical forms of treatment such as the Hall Technique,^44^ these findings raise the issue of whether this is an appropriate management approach in the twenty-first century. However, alternative approaches for caries management may only be considered if children attend regularly, and early enough, in the disease process when preventive,^42^ and simple restorative techniques have a strong evidence base.^44^ This issue needs to be addressed jointly by the profession and society, otherwise we continue to have ‘failure on all fronts’.^45^

### Strengths and limitations

These cross-sectional data provide a useful picture of the distribution of sedation across England; however, as the data only relate to courses of care involving sedation and they are not available along with the full details of clinical care provided, there are limits to their interpretation. Past research involving dental uptake and deprivation status in South East London suggests that whereas there is an inverse correlation amongst children, amongst adults the correlation is close to zero, which may be associated with the moderating effect of the NHS payment system, together with supplemental private dental care provision for adults. As already outlined, however, this analysis does not include private dental care; likewise it does not include hospital admissions for treatment under sedation and local anaesthesia or GA. Both are required to build a clear picture of the population in future and shape health services.

### Implications for research

There is need for further research on the relationship between social determinants and the use of sedation services. This will require more in-depth data on patients and their circumstances in relation to the timing and nature of dental care, including the potential for more preventative care. It will also provide an understanding on the level influence patient factors have on diseases. The importance of large data set analysis and learning is stressed within health services research. Data on treatments provided, as well as further information on NHS payment status, gender, risk status, smoking and dental anxiety, should be collected in future to permit in-depth and multivariate analysis to ascertain whether the relationships indicated in age and deprivation are confounded by other factors. Finally, linking primary-care and hospital data would also provide evidence of whether children are accessing both systems during their dental career.

In conclusion, patients receive sedation in support of NHS primary dental care across the life-course and social spectrum. The uptake of care parallels the social gradient, with exception of older people; however, geographical inequalities in access to primary dental care sedation services appear to be present within the system.

## Figures and Tables

**Table 1:** NHS patients and courses involving sedation in England, by age band, 2012–2014

*Age band (years)*	*2012/2013*					*2013/2014*				
	*No. of courses of care*	*%*	*No. of patients*	*%*	*Average per patient*	*Courses of care*	*%*	*Average per patient*	*No. of patients*	*%*
0–2	224	0.2	220	0.2	1.02	196	0.1	1.02	193	0.2
3–5	7,951	5.8	7,341	6.1	1.08	7,546	5.5	1.07	7,026	5.8
6–12	32,749	23.9	28,434	23.7	1.15	33,678	24.7	1.15	29,310	24.3
13–17	15,066	11.0	13,466	11.2	1.12	14,812	10.9	1.11	13,327	11.1
18–24	14,556	10.6	12,708	10.6	1.15	13,827	10.1	1.13	12,273	10.2
25–34	22,306	16.3	19,140	15.9	1.17	22,420	16.5	1.16	19,384	16.1
35–44	18,131	13.3	15,771	13.1	1.15	17,210	12.6	1.13	15,167	12.6
45–54	14,892	10.9	13,089	10.9	1.14	14,922	11.0	1.13	13,216	11
55–64	7,163	5.2	6,439	5.4	1.11	7,478	5.5	1.11	6,718	5.6
65–74	2,938	2.1	2,651	2.2	1.11	3,204	2.4	1.09	2,946	2.4
75+	839	0.6	776	0.6	1.08	970	0.7	1.07	908	0.8
Total	136,815	100	120,035	100	1.14	136,263	100	1.13	120,468	100

**Table 2:** NHS patients and courses involving sedation in England, by deprivation category, 2012–2014

*Age band*	*2012/2013*					*2013/2014*				
	*No. of courses of care*	*%*	*No. of patients*	*%*	*Average per patient*	*No. of courses of care*	*%*	*No. of patients*	*%*	*Average per patient*
Most deprived	42,980	31.4	37,056	29	1.16	39,980	29.3	34,925	29	1.14
2	32,963	24.1	28,942	22.4	1.14	30,523	22.4	27,032	22.4	1.13
3	24,238	17.7	21,548	18.6	1.12	25,031	18.4	22,360	18.6	1.12
4	18,251	13.3	16,521	15.3	1.1	20,476	15	18,425	15.3	1.11
Least deprived	14,691	10.7	13,577	12.2	1.08	16,034	11.8	14,691	12.2	1.09
Non-categorised	3,692	2.7	2,391	2.5	1.54	4,219	3.1	3,035	2.5	1.39
Total	136,815	100	120,035	100	1.14	136,263	100	120,468	100	1.13

**Table 3:** Average courses of care involving sedation by age group and NHS Band of care, 2012–2014

*2012/2013*						*2013/2014*				
*Patient age range (years)*	*No. of patients*	*Band 1 FP17s with sedation*	*Band 2 FP17s with sedation*	*Band 3 FP17s with sedation*	*Band 1 urgent FP17s with sedation*	*No. of patients*	*Band 1 FP17s with sedation*	*Band 2 FP17s with sedation*	*Band 3 FP17s with sedation*	*Band 1 urgent FP17s with sedation*
0–2	220	0.12	0.89	0	0	193	0.26	0.75	0	0.01
3–5	7,341	0.04	1.03	0	0	7,026	0.06	1.01	0	0
6–12	28,434	0.05	1.09	0	0	29,310	0.06	1.08	0	0
13–17	13,466	0.06	1.03	0.03	0	13,327	0.06	1.02	0.03	0
18–24	12,708	0.05	0.99	0.08	0	12,273	0.05	0.98	0.07	0
25–34	19,140	0.05	0.99	0.1	0	19,384	0.05	0.99	0.09	0
35–44	15,771	0.05	0.95	0.13	0	15,167	0.05	0.94	0.12	0
45–54	13,089	0.05	0.92	0.15	0	13,216	0.06	0.92	0.13	0
55–64	6,439	0.05	0.89	0.14	0	6,718	0.06	0.89	0.13	0.01
65–74	2,651	0.05	0.9	0.13	0	2,946	0.07	0.86	0.11	0.01
75+	776	0.06	0.86	0.1	0.03		0.1	0.85	0.08	0.02
Total	120,035	0.05	1	0.07	0	120,468	0.06	0.99	0.06	0

Note NHS Bands of care^46^ NHS payment system in England from April 2006. For further information see NHS Choices—http://www.nhs.uk/nhsengland/aboutnhsservices/dentists/pages/nhs-dental-charges.aspx.

Urgent treatment covers examination, X-rays and treatments such as dressings, re-cementing crowns and up to two tooth extractions and one tooth restoration.

Band 1 involves assessment of a patient, which includes diagnosis, treatment planning and maintenance, examination, X-rays, scale and polish, preventative work and minor changes to dentures.

Band 2 constitutes any Band 1 items plus treatment that does not involve laboratory work, for example, tooth restoration, root canal treatment, tooth extraction and periodontal treatment.

Band 3 involves complex treatment that includes a laboratory element, for example,^24^ bridges, crowns and dentures, in addition to any Band 1 or 2 level of care.
